# EOG Artifact Removal from EEG Using a Gated WGAN with Multi-Scale and Multi-Domain Feature Extraction

**DOI:** 10.3390/brainsci16101124

**Published:** 2026-10-17

**Authors:** Songyun Xie, Xianghui Liu, Xiangming Wang, Xinzhou Xie, Hao Tang

**Affiliations:** School of Electronics and Information, Northwestern Polytechnical University, Xi’an 710072, China; xianghuiliu@mail.nwpu.edu.cn (X.L.); wxming@mail.nwpu.edu.cn (X.W.); xinzhxie@nwpu.edu.cn (X.X.); haotang@mail.nwpu.edu.cn (H.T.)

**Keywords:** EEG, EOG, Wasserstein generative adversarial network, artifact removal, multi-scale feature extraction

## Abstract

**Background**: Electroencephalogram (EEG) signals provide important information for brain–computer interfaces (BCIs) and the diagnosis of neurological disorders but are susceptible to electrooculographic (EOG) artifacts. Traditional methods often rely on multichannel data or relatively long windows, limiting online applicability. Deep learning enables single-channel end-to-end artifact removal, but existing methods may not fully exploit features in the time and frequency domains and may unnecessarily modify relatively clean EEG. **Methods**: We developed a Wasserstein generative adversarial network (WGAN) integrating reconstruction loss, multi-domain and multi-scale feature extraction, and a gating unit. Reconstruction loss improved waveform reconstruction, yielding a reconstruction-loss-assisted artifact-removal WGAN (rarWGAN). Parallel branches with different kernel sizes extracted multi-scale features, while a multi-domain module combined temporal features with spectral features extracted using complex-valued convolutions. Integrating both modules into rarWGAN produced MDS-rarWGAN. Based on discriminator scores, gating routed inputs classified as contaminated to the generator and retained the others unchanged. **Results**: On EEGdenoiseNet, MDS-rarWGAN yielded relative root mean square errors (RRMSEs) of 0.189 ± 0.028 and 0.183 ± 0.027 in the time and frequency domains, respectively, with a correlation coefficient of 0.955 ± 0.013. Compared with rarWGAN, RRMSEs decreased by 23.2% and 32.5%, respectively, and the correlation coefficient increased by 1.2%. The gating unit yielded false positive and false negative rates of 1.47% and 0.12%, respectively. For EEG containing artifacts, no statistically significant differences in time domain RRMSE were detected with and without gating (all *p* > 0.05). On BCI Competition IV 2a, reconstruction metrics measured agreement with a reference derived from independent component analysis (ICA), as artifact-free EEG ground truth was unavailable. Using the same EEGNet classifier, EEG processed by gated-MDS-rarWGAN achieved the highest mean motor imagery classification accuracy among the evaluated deep learning methods. **Conclusions**: The method suppresses EOG artifacts, reduces unnecessary processing of signals without evident artifacts, and supports motor imagery decoding, offering a feasible preprocessing approach.

## 1. Introduction

Electroencephalogram (EEG) signals reflect neural activity in the brain and constitute an important source of information for clinical diagnosis and neuroscience research [1]. Noninvasive brain–computer interfaces (BCIs) acquire and decode EEG signals to translate brain activity into control commands for external devices [2]. Therefore, EEG signal quality directly affects the accuracy of neural information extraction and the reliability of BCI systems [3]. However, during EEG acquisition, the signals are inevitably contaminated by noncerebral physiological activity and environmental noise, collectively referred to as artifacts. Common artifacts include electromyographic (EMG), electrooculographic (EOG), and electrocardiographic (ECG) artifacts, as well as electrode and environmental noise [4]. Because some artifacts overlap with EEG signals in both the time and frequency domains, conventional filtering alone cannot readily suppress them while fully preserving neural information [5]. In this study, EOG artifacts collectively refer to EEG contamination arising from eye blinks and eye movements, including saccades. Horizontal eye movements are primarily reflected in horizontal EOG (HEOG), whereas vertical eye movements and blinks are more prominent in vertical EOG (VEOG) [6]. EOG artifacts often have large amplitudes and substantial energy at low frequencies, potentially interfering with EEG feature extraction and subsequent analyses. Effective suppression of EOG artifacts with minimal loss of useful EEG information is therefore important for improving the reliability of EEG analysis and BCI systems [7].

Conventional approaches to EOG artifact removal include regression, adaptive filtering, wavelet transforms, and blind source separation methods such as independent component analysis (ICA) [8]. ICA decomposes multichannel EEG signals into statistically independent components. Artifact-related components are then identified manually or with automated classifiers such as ICLabel and subsequently removed [9,10]. ICA allows individual components to be inspected, but its performance depends on the number of channels and how well the assumptions of statistical independence and stationarity are satisfied [9]. Errors in component classification may leave residual artifacts or lead to the removal of neural components [10]. Depending on the method, conventional approaches may require reference EOG channels, multichannel EEG recordings, manually selected parameters, or sufficiently long data segments. These requirements can limit their applicability to single-channel and online applications.

The release of benchmark datasets such as EEGdenoiseNet has facilitated the development and evaluation of end-to-end deep learning methods for EEG artifact removal [11]. Convolutional neural networks (CNNs) and recurrent neural networks (RNNs) can learn nonlinear mappings directly from contaminated EEG signals to their clean counterparts, reducing the need for handcrafted features [11,12]. For example, IC-U-Net uses a U-Net trained on mixtures of brain and nonbrain sources obtained through ICA to automatically remove multiple types of EEG artifacts [13]. DuoCL uses two convolutional branches with different kernel sizes to extract morphological features and a long short-term memory network to capture temporal dependencies [14]. These studies highlight the importance of network architecture and temporal feature extraction for EEG reconstruction.

Generative adversarial networks (GANs) use adversarial training to reduce distributional differences between reconstructed EEG signals and clean reference signals, providing an alternative approach to EEG artifact removal. AR-WGAN uses Wasserstein adversarial learning for automatic end-to-end EEG artifact removal [15]. EEGANet uses a GAN to remove ocular artifacts without requiring reference EOG channels or eye-blink detection during application [16]. AnEEG incorporates long short-term memory layers into a GAN to remove multiple artifacts caused by eye and muscle activities [17]. AGO-CycleGAN combines attention-guided generators with a CycleGAN framework to restore single-channel EEG signals contaminated by motion artifacts [18]. These studies demonstrate the applicability of generative adversarial learning to the removal of ocular, muscle, and motion artifacts from EEG signals.

Despite these advances, several issues remain in EOG artifact removal from single-channel EEG. First, adversarial loss alone mainly constrains the overall distribution of the generated signals and does not directly penalize pointwise reconstruction errors. Second, EEG and EOG signals contain useful information at different temporal scales and in both the time and frequency domains. Previous studies have explored multi-scale structures or spectral information [3,19], but these complementary features can be further integrated for EEG reconstruction. Third, applying the same denoising process to all input signals may unnecessarily modify EEG signals without evident artifacts. Recent work has used contamination estimation to guide selective processing, with training on EEGdenoiseNet mixtures and evaluation on independent recordings [20].

Accordingly, this study aims to develop an EOG artifact removal framework for single-channel EEG that improves EEG reconstruction while reducing unnecessary modification of relatively clean EEG signals. To this end, we propose gated-MDS-rarWGAN. The main contributions of this study are as follows:

1. Adversarial and reconstruction losses are jointly optimized to reduce distributional differences and pointwise reconstruction errors, respectively.
2. Parallel convolutional branches with different kernel sizes extract multi-scale temporal features, while a multi-domain module combines temporal features with spectral features extracted using complex-valued convolutions.
3. A gating unit uses discriminator scores and a threshold selected on validation data to route inputs classified as contaminated to the generator and retain the others unchanged. This design avoids the need for a separate artifact detection network and reduces unnecessary processing.
4. Experiments on EEGdenoiseNet evaluate reconstruction performance, the contributions of the model components, and gating behavior. Experiments on real motor imagery EEG from BCI Competition IV 2a examine agreement with an ICA-derived reference and downstream classification performance.

## 2. Materials and Methods

**Figure 1:**
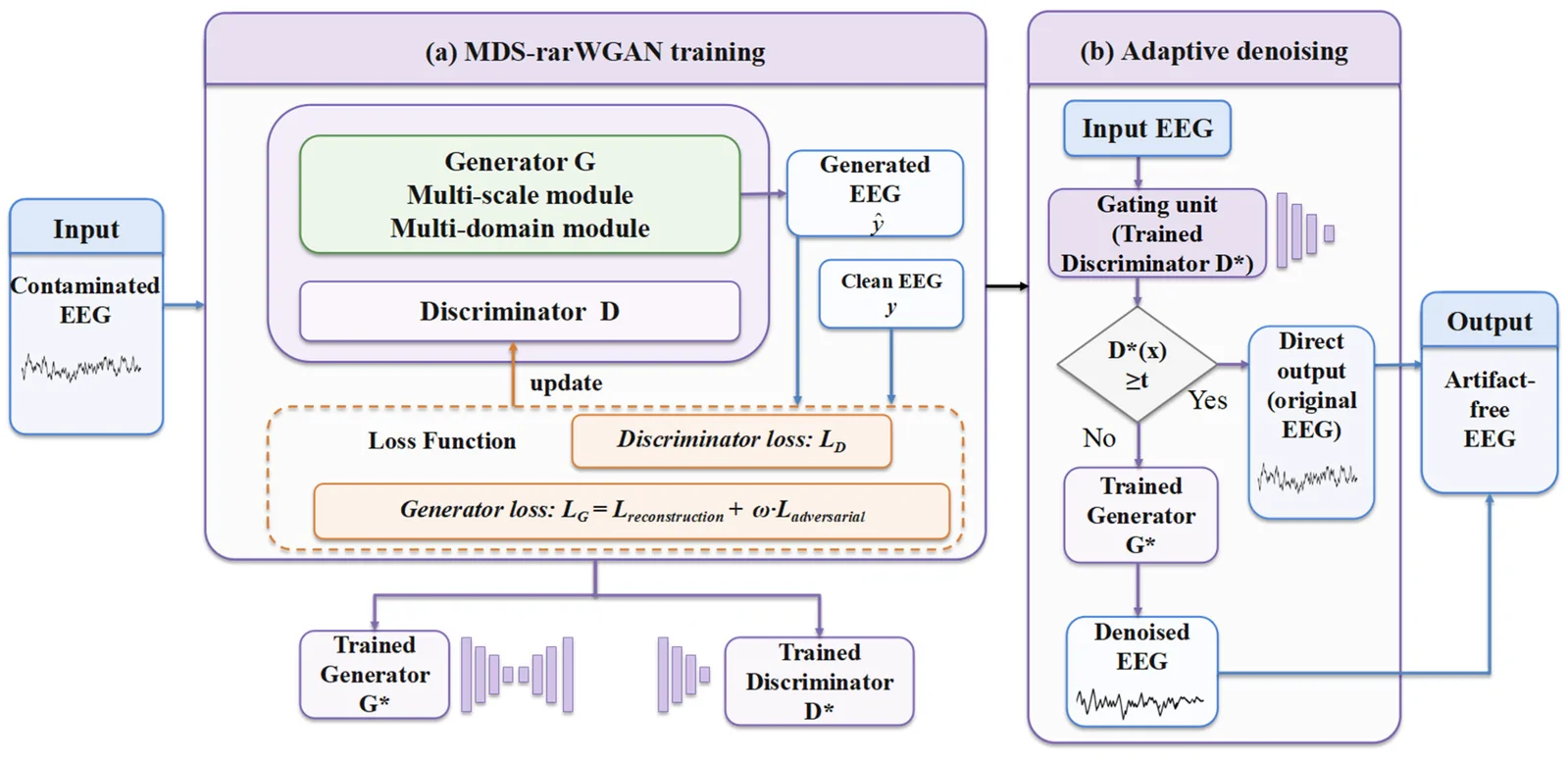
Overall framework of the proposed gated-MDS-rarWGAN: (**a**) training of MDS-rarWGAN; (**b**) adaptive denoising based on the trained discriminator. The “*” denotes the trained models.

As illustrated in Figure 1, the proposed gated-MDS-rarWGAN comprises two stages: (a) MDS-rarWGAN training and (b) adaptive denoising. Let *x* denote an EEG segment contaminated by EOG artifacts and *y* the corresponding clean EEG segment. The objective of EEG artifact removal is to learn a mapping from artifact-contaminated EEG to clean EEG:

(1) $$\hat{y}=G(x)$$

where G(·) denotes the generator and $\hat{y}$ is the reconstructed EEG signal. During adaptive denoising, a gate based on the trained discriminator determines whether each input is processed by the generator or retained unchanged.

### 2.1. rarWGAN: WGAN with Reconstruction Loss for Artifact Removal

GANs learn to approximate the target data distribution through adversarial training between a generator and a discriminator [21]. The Wasserstein generative adversarial network (WGAN) uses the Wasserstein distance to measure the discrepancy between the generated and real data distributions, which can improve training stability and alleviate problems such as vanishing gradients and mode collapse [22]. However, the adversarial objective primarily encourages the distribution of the generated EEG signals to resemble that of the clean EEG signals. It does not directly penalize pointwise differences between the generated EEG signals and their corresponding clean targets and may therefore be insufficient for accurately reconstructing local waveform details and amplitudes. To improve pointwise reconstruction accuracy, mean squared error (MSE) is incorporated into the WGAN generator objective as a reconstruction loss. The reconstruction loss is defined as:

(2) $$L_{reconstruction}=\frac{1}{n}\sum_{i=1}^{n}{\left(y_{i}-{\hat{y}}_{i}\right)}^{2}$$

where $y_{i}$ and ${\hat{y}}_{i}$ denote the values of the target clean EEG signal and the generated EEG signal at sample *i*, respectively, and *n* is the number of sampling points. However, when used alone, MSE focuses on pointwise agreement rather than explicit distribution matching and may lead to overly smooth reconstructions. Therefore, the generator is jointly optimized using the reconstruction and adversarial losses:

(3) $$L_{G}=L_{reconstruction}+\omega \cdot L_{\mathrm{adversarial}}$$

Here, $L_{\mathrm{adversarial}}$ denotes the original adversarial loss of the WGAN generator, and *ω* is its weighting coefficient. The candidate values 0, 0.00001, 0.00005, 0.0001, 0.0005, 0.001, 0.005, 0.01, 0.05, 0.1, 0.5, and 1 were evaluated for rarWGAN on EEGdenoiseNet. A value of 0.001 was selected because it yielded the lowest mean time-domain and frequency-domain RRMSEs among the evaluated values while maintaining a high CC.

### 2.2. MDS-rarWGAN: Multi-Domain and Multi-Scale rarWGAN

The rarWGAN generator relies primarily on time-domain convolutions with a single kernel size for feature extraction. Multi-domain and multi-scale feature extraction modules are added to incorporate spectral information and temporal features at multiple scales, forming MDS-rarWGAN.

**Figure 2:**
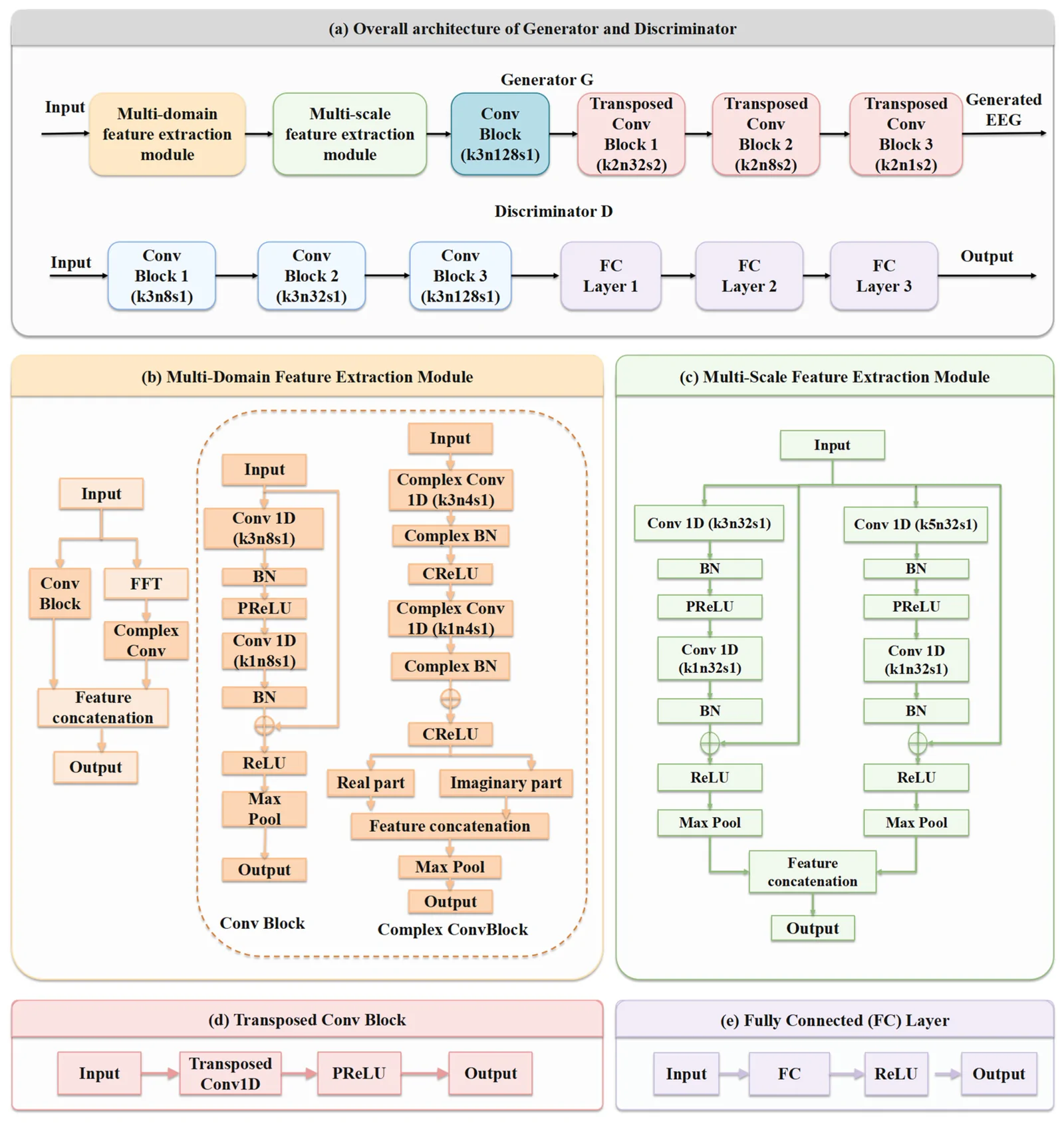
Architecture of MDS-rarWGAN: (**a**) overall architecture of the generator and discriminator; (**b**) multi-domain feature extraction module; (**c**) multi-scale feature extraction module; (**d**) transposed convolution block; and (**e**) fully connected layer. Here, *k*, *n*, and *s* denote the kernel size, number of output channels, and stride, respectively.

The detailed architectures of the generator and discriminator are shown in Figure 2. The generator comprises a multi-domain feature extraction module, a multi-scale feature extraction module, a convolutional block (ConvBlock), and three transposed convolution blocks. Inspired by residual networks (ResNets) [23], the ConvBlock employs residual connections and consists of convolutional layers, batch normalization (BN) layers, activation functions, and a max pooling layer. The transposed convolution blocks progressively restore the temporal resolution through upsampling and ultimately produce a reconstructed signal with the same length as the input. The discriminator comprises three convolutional blocks and three fully connected layers and outputs a score.

#### 2.2.1. Multi-Domain Feature Extraction Module

The multi-domain feature extraction module is shown in Figure 2b and consists of a time-domain branch and a frequency-domain branch. The time-domain branch uses a conventional ConvBlock to extract temporal features. The frequency-domain branch transforms the input into a complex spectral representation and uses a complex ConvBlock to extract spectral features. The outputs of the two branches are concatenated along the channel dimension to form a joint temporal–spectral representation. In the frequency-domain branch, an *N*-point fast Fourier transform (FFT) is applied along the sample dimension, with *N* equal to the input segment length [24]. The forward transform uses the unscaled discrete Fourier transform (DFT) convention, and all *N* complex coefficients are retained for feature extraction. For a sequence *x*[*n*], the transform is defined as:

(4) $$X\left[k\right]=\sum_{n=0}^{N-1}x\left[n\right]e^{-j\frac{2\pi }{N}kn},\;k=0,1,\ldots ,N-1$$

Here, *j* denotes the imaginary unit. The transform produces *N* complex frequency coefficients containing both magnitude and phase information. For a real-valued input, the coefficients satisfy conjugate symmetry:

$$X\left[N-k\right]=X\left[k\right]*,\;k=1,\ldots ,N-1$$

where the superscript * denotes complex conjugation. A complex convolutional neural network is used to process the real and imaginary components jointly [25]. For a complex feature map *M* = *M_R_* + *jM_I_* and a complex convolutional kernel *K* = *K_R_* + *jK_I_*, complex convolution is expressed as

(5) $$M*K=\left(M_{R}+jM_{I}\right)*\left(K_{R}+jK_{I}\right)=\left(M_{R}K_{R}-M_{I}K_{I}\right)+j\left(M_{R}K_{I}+M_{I}K_{R}\right)$$

Here, * denotes convolution. The coupled operations allow information from both components to contribute to the extracted features. Complex batch normalization treats the real and imaginary components of each feature as a two-dimensional vector:

$$u=\left[\begin{array}{l} u_{R}\\ u_{I} \end{array}\right]$$

Its covariance matrix is:

(6) $$V=\left[\begin{array}{l} Cov(u_{R},u_{R})\;Cov(u_{R},u_{I})\\ Cov(u_{I},u_{R})\;Cov(u_{I},u_{I}) \end{array}\right]$$

The centered vector is whitened using the inverse square root of the covariance matrix, followed by a learnable affine transformation:

(7) $$\begin{array}{l} \hat{u}=V^{-1/2}(u-E\left[u\right])\\ y=\Gamma \hat{u}+\beta \end{array}$$

Here, Γ and *β* are the learnable scale and shift parameters. This normalization accounts for both the component variances and the covariance between the real and imaginary components. The complex rectified linear unit (CReLU) applies ReLU separately to the real and imaginary components:

(8) CReLU(z)=ReLU(Re(z))+jReLU(Im(z))

The complex ConvBlock follows the residual structure of the conventional ConvBlock, with complex convolution, complex batch normalization, and CReLU replacing the corresponding real-valued operations. After the residual block, the real and imaginary feature maps are concatenated along the channel dimension before max pooling. This converts the complex features into a real-valued representation compatible with the subsequent generator layers while retaining information from both components.

#### 2.2.2. Multi-Scale Feature Extraction Module

As shown in Figure 2c, the multi-scale feature extraction module consists of two parallel convolutional branches with different receptive fields. Branch 1 uses a convolutional kernel of size 1 × 3, whereas Branch 2 uses a kernel of size 1 × 5. Padding sizes of 1 and 2 are applied along the temporal dimension in Branches 1 and 2, respectively, to preserve the temporal length of the feature maps. Each branch incorporates residual connections, activation functions, and max pooling. The branch outputs are then concatenated along the channel dimension to form a joint multi-scale representation. This module extracts EEG and EOG features over different temporal ranges to represent artifacts with varying temporal patterns.

### 2.3. Adaptive Denoising with Gating

The gating unit determines whether an input EEG signal should be processed by the generator. For an input signal *x*, the output after gating is defined as:

(9) $$Output_{EEG}=g(x)\cdot G*(x)+(1-g(x))\cdot x$$

where *g*(*x*) is the binary gating decision and G*(·) denotes the trained MDS-rarWGAN generator. When *g*(*x*) = 1, the input is processed by the generator; when *g*(*x*) = 0, the original input is retained. The gating decision is based on the scalar score produced by the trained WGAN discriminator rather than probability. After model training, clean and contaminated EEG signals in the validation set are passed through the discriminator to determine the gating threshold. The binary gating function is defined as:

(10) $$g(x)=\left\{\begin{array}{l} 0,\;D*(x)\geq t\\ 1,\;D*(x)<t \end{array}\right.$$

where D*(·) denotes the trained discriminator and *t* is the gating threshold. This design reuses the discriminator as the gating unit without introducing a separate artifact detection network, as illustrated in Figure 1b.

The threshold *t* is determined as follows:

1. Validation set: clean EEG data obtained from Pure EEG and artifact-contaminated EEG data generated by mixing Pure EEG with Pure EOG at signal-to-noise ratios (SNRs) ranging from −7 to 2 dB.
2. Score calculation: All samples in the validation set are passed through the trained WGAN discriminator, and the D*(*x*) score of each sample is recorded.
3. Candidate threshold generation: One hundred equally spaced candidate thresholds *t* are generated between *min*(D*(*x*)) and *max*(D*(*x*)). For each threshold, the gate classifies a sample with D*(*x*) ≥ *t* as artifact-free EEG and a sample with D*(*x*) < *t* as artifact-contaminated EEG.
4. Performance evaluation: The classification performance is calculated for each candidate threshold. Accuracy is used when the two classes are balanced, whereas the F1 score is used when they are imbalanced.
5. Threshold selection: The candidate yielding the highest validation performance is selected as the final gating threshold *t*.

### 2.4. Evaluation Metrics

Artifact removal performance was evaluated using the relative root mean square error (RRMSE) in the time and frequency domains and the correlation coefficient (CC). The time domain RRMSE is defined as:

(11) $$\mathrm{RRMSE}\text{\_}t=\frac{RMS\left(\hat{y}-y\right)}{RMS\left(y\right)}$$

where *y* denotes the reference EEG signal and $\hat{y}$ denotes the denoised output. The root mean square (RMS) of a signal *z* containing *N* samples is calculated as:

(12) $$RMS\left(z\right)=\sqrt{\frac{1}{N}\sum_{i=1}^{N}z_{i}^{2}}$$

The frequency domain RRMSE is defined as:

(13) $$\mathrm{RRMSE}\text{\_}f=\frac{RMS\left(PSD(\hat{y})-PSD(y)\right)}{RMS\left(PSD(y)\right)}$$

where *PSD*(·) denotes the power spectral density [26]. PSD was estimated using the Welch method implemented in SciPy version 1.10.1, with a periodic Hann window of 512 samples, an overlap of 256 samples (50%), and a 512-point FFT. The sampling frequency used in the calculation was 256 Hz, corresponding to a window duration of 2 s. Because the EEG and EOG signals were bandpass filtered at 1–80 Hz and 0.3–10 Hz, respectively, the PSD comparison was restricted to 0–80 Hz. The CC is defined as:

(14) $$\mathrm{CC}=\frac{Cov(\hat{y},y)}{\sqrt{Var(\hat{y})Var(y)}}$$

## 3. Experiments and Results on the EEGdenoiseNet Dataset

EEGdenoiseNet is a benchmark dataset containing 4514 clean EEG segments derived from recordings of 52 participants and 3400 ocular artifact segments extracted from horizontal and vertical EOG recordings in several public datasets [11]. Because the dataset does not provide separate labels for eye blinks, saccades, and other eye movements, the ocular artifact segments were collectively treated as EOG artifacts. Following the original EEGdenoiseNet benchmark protocol, 3400 clean EEG segments and 3400 EOG segments were split into training, validation, and test subsets in an 8:1:1 ratio. This division was performed at the segment level before generating the semi-synthetic mixtures. Mixtures were generated separately within each subset, and no EEG or EOG source segment was shared across these subsets. The released segment files do not provide the participant or original recording label corresponding to each segment; therefore, a reliable division based on participant or recording identity could not be reconstructed. The contaminated EEG was generated by randomly mixing the clean EEG and EOG segments at ten SNR levels ranging from −7 to 2 dB, with the corresponding clean EEG segments serving as the ground truth. Each segment contained 512 samples, and the contaminated input and clean target were normalized using the standard deviation of the contaminated input.

MDS-rarWGAN incorporates both the multi-domain and multi-scale feature extraction modules. Multi-domain-rarWGAN retains only the multi-domain feature extraction module, with the multi-scale module replaced by a conventional convolutional block. Multi-scale-rarWGAN retains only the multi-scale feature extraction module, with the multi-domain module replaced by a conventional convolutional block. rarWGAN uses conventional convolutional blocks without either of the proposed feature extraction modules. WGAN uses the same network architecture as rarWGAN but is trained without the reconstruction loss.

### 3.1. Effect of Reconstruction Loss

To evaluate the contribution of reconstruction loss to EEG artifact removal, rarWGAN was compared with WGAN and four baseline networks. The main hyperparameters of the fully connected neural network (FCNN), RNN, Simple CNN, and Complex CNN [11] are summarized in Table 1. For a fair comparison, WGAN and rarWGAN used the same number of training epochs, learning rate, and batch size as Simple CNN and Complex CNN, namely 50 epochs, a learning rate of 5 × 10^−5^, and a batch size of 50.

**Figure 3:**
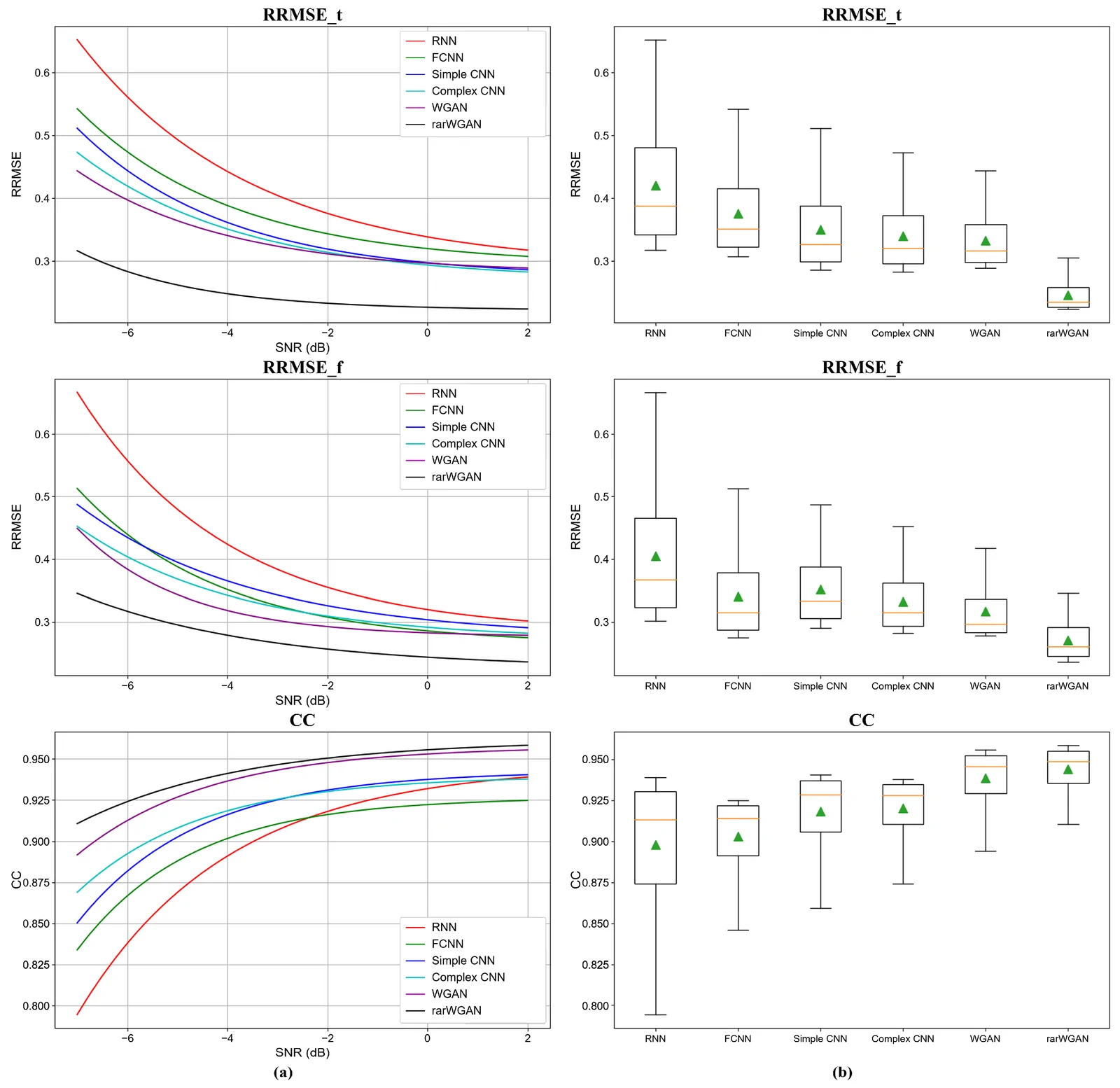
EOG artifact removal performance of different methods. (**a**) Time domain RRMSE, frequency domain RRMSE, and CC at different SNR levels; (**b**) boxplots showing the overall distributions of the three metrics.

Figure 3 shows the time domain RRMSE, frequency domain RRMSE, and CC of each method at different SNR levels and their overall distributions. As the SNR increased, the RRMSEs of all methods decreased, while the CC increased. This indicates that EEG recovery became easier as the artifacts became weaker. The differences between the models were larger at low SNRs. RNN showed the largest changes in performance across SNR levels. rarWGAN achieved the lowest RRMSEs and the highest CC at all SNR levels, with smaller changes in performance across SNR levels. These results show that combining adversarial loss with reconstruction loss improves denoising performance across different SNR levels.

Compared with WGAN, rarWGAN reduced the mean RRMSE from 0.333 to 0.246 in the time domain and from 0.317 to 0.271 in the frequency domain, while increasing the mean CC from 0.939 to 0.944. The distributions of the two RRMSEs were also more concentrated.

### 3.2. Effects of the Multi-Scale and Multi-Domain Modules

**Figure 4:**
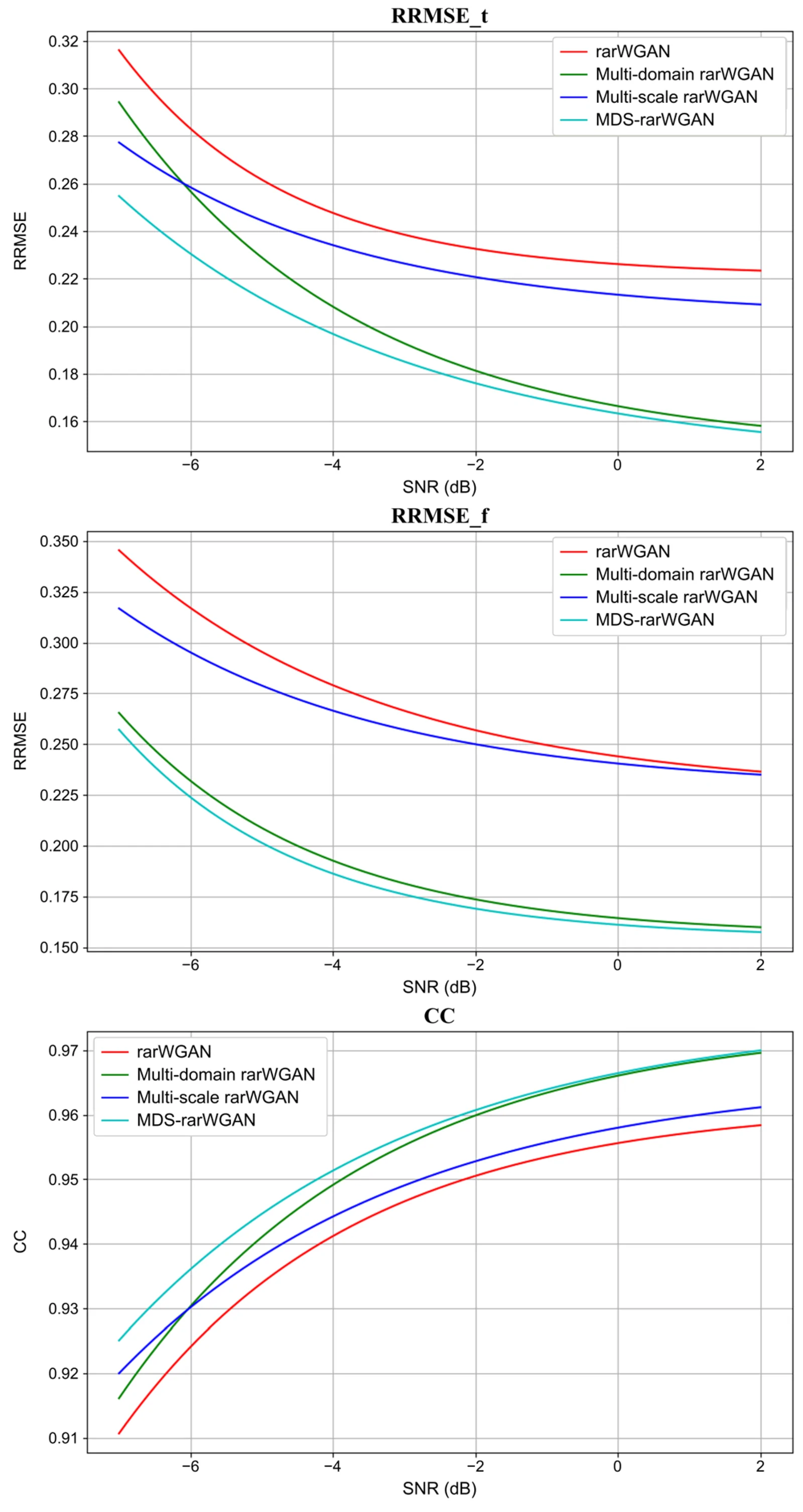
Ablation study of the multi-scale and multi-domain modules at different SNR levels.

Figure 4 compares rarWGAN with variants incorporating one or both modules at different SNR levels. Adding either module alone reduced both RRMSEs and increased CC relative to rarWGAN. The multi-scale module provided better reconstruction in the time domain at low SNRs, whereas the multi-domain module produced a greater reduction in frequency-domain RRMSE. When both modules were combined, MDS-rarWGAN achieved the lowest RRMSEs in both the time and frequency domains and the highest or nearly highest CC across all SNR levels.

To further evaluate its EOG artifact removal performance, MDS-rarWGAN was compared with RNN, FCNN, Simple CNN, Complex CNN, WGAN, and representative recent methods, including DuoCL [14], GCTNet [27], and MSCGRU [28]. All comparator models were trained and evaluated in this study using the same data partitions, preprocessing, and contaminated test inputs as MDS-rarWGAN. The three metrics were calculated using a common evaluation program, with the same PSD estimation settings used for frequency-domain RRMSE. Training hyperparameters were configured for each model. Table 2 presents the RRMSEs and CCs of all models on the EEGdenoiseNet test set using the segment-level split described above. MDS-rarWGAN achieved the lowest mean RRMSEs of 0.189 ± 0.028 and 0.183 ± 0.027 in the time and frequency domains, respectively, and the highest mean CC of 0.955 ± 0.013 among the compared methods. Compared with the recent methods, its time-domain RRMSE was 29.2% lower than that of MSCGRU, its frequency-domain RRMSE was 25.6% lower than that of GCTNet, and its CC was 1.2% higher than that of GCTNet. The RRMSE and CC values in Table 2 are reported as mean ± standard deviation (SD). Approximate Cohen’s *d*_av_ values were calculated from the rounded means and SDs by subtracting the comparator mean from the MDS-rarWGAN mean and dividing by the average of their SDs. Negative effect sizes for RRMSE and positive effect sizes for CC favor MDS-rarWGAN. These approximate effect sizes were used to describe the magnitude of differences between methods, rather than to assess statistical significance.

Table 2 also quantifies the effects of the multi-scale and multi-domain modules. When added separately to rarWGAN, the multi-scale module reduced the two RRMSEs by 6.5% and 3.7%, respectively, whereas the multi-domain module reduced them by 18.7% and 30.6%. Combining both modules in MDS-rarWGAN reduced the RRMSEs relative to rarWGAN by 23.2% and 32.5%, respectively, and increased the CC from 0.944 to 0.955.

**Figure 5:**
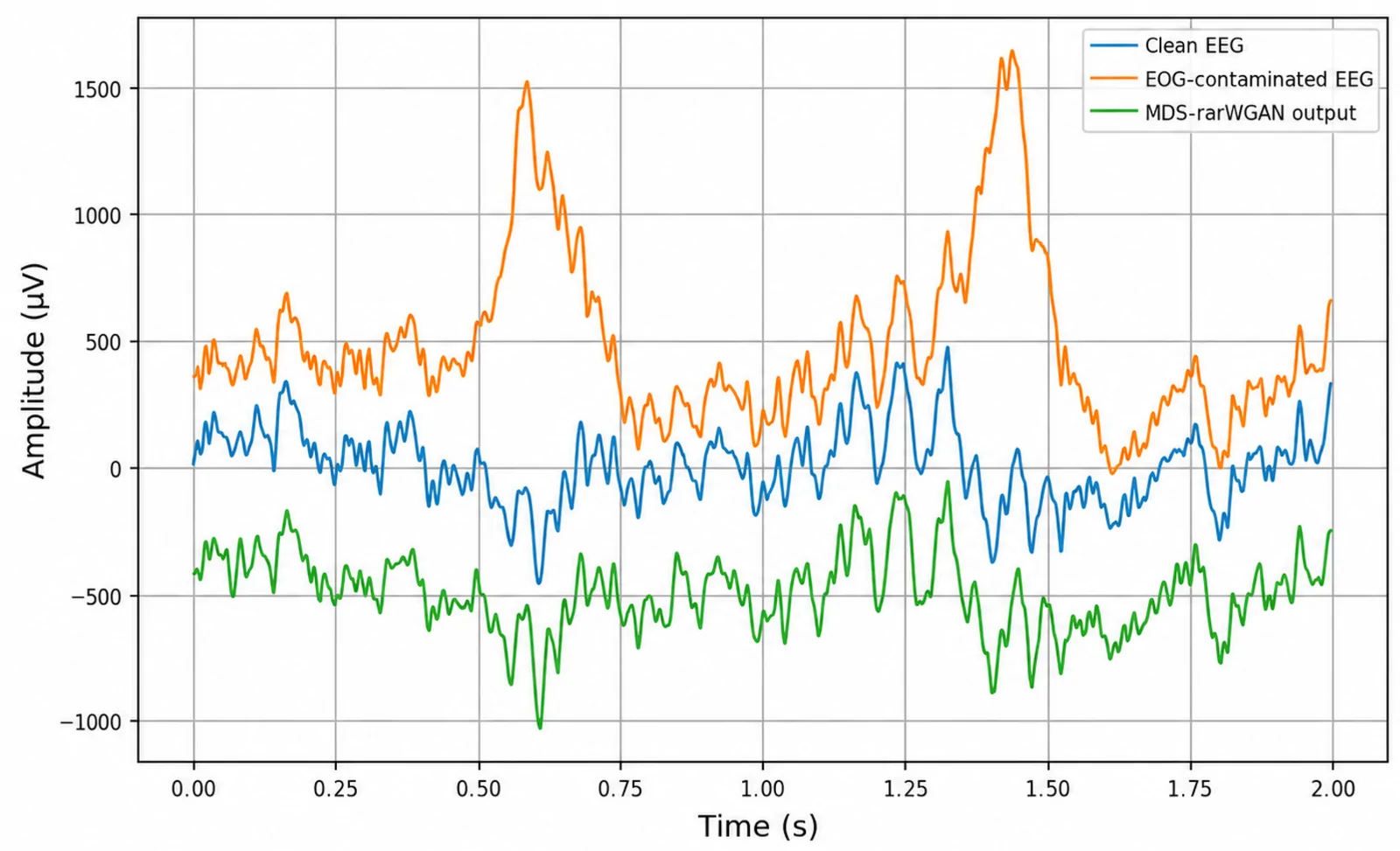
Comparison of clean EEG, EOG-contaminated EEG, and the MDS-rarWGAN output on the EEGdenoiseNet dataset.

Figure 5 illustrates a representative example of waveform reconstruction on EEGdenoiseNet, including the clean EEG, the EOG-contaminated EEG, and the MDS-rarWGAN output. For clearer visualization, constant vertical offsets were applied to the EOG-contaminated EEG and MDS-rarWGAN output; therefore, their displayed amplitude values do not represent the original absolute values. The EOG-contaminated EEG exhibits pronounced large-amplitude deflections, particularly at approximately 0.6 and 1.45 s. After artifact removal, the MDS-rarWGAN output closely follows the waveform morphology of the clean EEG while substantially suppressing these ocular-artifact components.

To evaluate the computational efficiency of MDS-rarWGAN, Table 3 compares the parameter counts and average processing time per segment. The models were implemented in PyTorch version 2.3.1, and the experiments were conducted on Ubuntu 18.04 LTS using an NVIDIA GeForce RTX 3090 GPU. The average processing time was measured for the generator forward pass with a batch size of 1 after the input tensor had been loaded onto the GPU. This measurement included the FFT operation in the multi-domain module. MDS-rarWGAN contained 66.90 K parameters and had an average generator inference time of 1.4026 ms per 2-s single-channel EEG segment. The low computational cost suggests the potential of the model for future online implementation.

### 3.3. Effects of the Gating Unit

The gating unit was applied to the four baseline models and MDS-rarWGAN. In addition to the original 10 SNR levels ranging from −7 to 2 dB, artifact-free EEG segments (SNR = +∞) were included to represent clean inputs. Here, “artifact-free EEG” refers to the benchmark clean EEG segments without added EOG contamination. The validation set used for threshold selection contained 340 clean EEG segments and 3400 EOG-contaminated EEG segments. Because the classes were imbalanced, the threshold was selected by maximizing the validation F1 score. For gated-MDS-rarWGAN, the selected gating threshold was *t* = −24.6.

**Figure 6:**
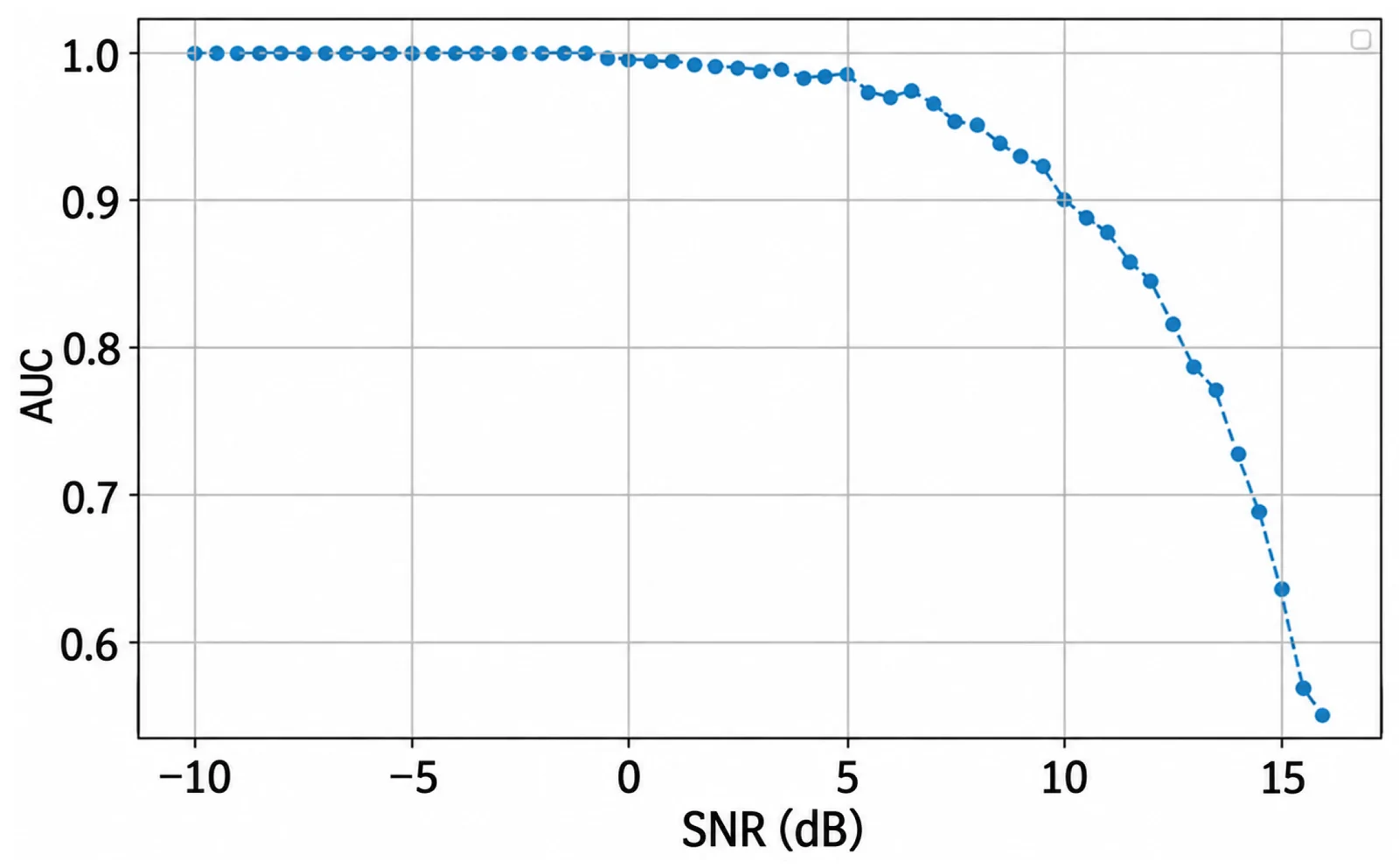
AUC of discriminator for detecting EOG artifacts at different SNR levels.

Figure 6 shows how the area under the receiver operating characteristic curve (AUC) of the discriminator varied with the SNR of the input signals. As the SNR increased, the AUC gradually decreased because weaker artifacts reduced the differences between EEG signals with and without artifacts. Nevertheless, throughout the SNR range of −7 to 2 dB, the discriminator maintained a high AUC, which was 0.99 at 2 dB. For gated-MDS-rarWGAN, 335 of the 340 clean test segments bypassed the generator, while five were incorrectly routed to it. Of the 3400 EOG-contaminated test segments, 3396 were routed to the generator and four were incorrectly bypassed. With EOG-contaminated EEG segments defined as the positive class, the sensitivity, specificity, precision, and F1 score were 99.88%, 98.53%, 99.85%, and 99.87%, respectively. The corresponding false positive and false negative rates were 1.47% and 0.12%. In the highest SNR interval of 1–2 dB, three of the 364 contaminated segments were incorrectly bypassed, corresponding to a false negative rate of 0.82%.

Table 4 compares the time-domain RRMSE of each model on EOG-contaminated EEG without and with gating. Both conditions were evaluated using the same 3400 test segments. The RRMSE values were compared using two-sided paired t-tests, with a significance level of 0.05. For MDS-rarWGAN, the corresponding RRMSE values were 0.189 ± 0.028 and 0.190 ± 0.026, respectively (*p* = 0.894). The five reported *p*-values were additionally adjusted using the Holm method. All adjusted *p*-values were 1.000, and no statistically significant differences were detected.

**Figure 7:**
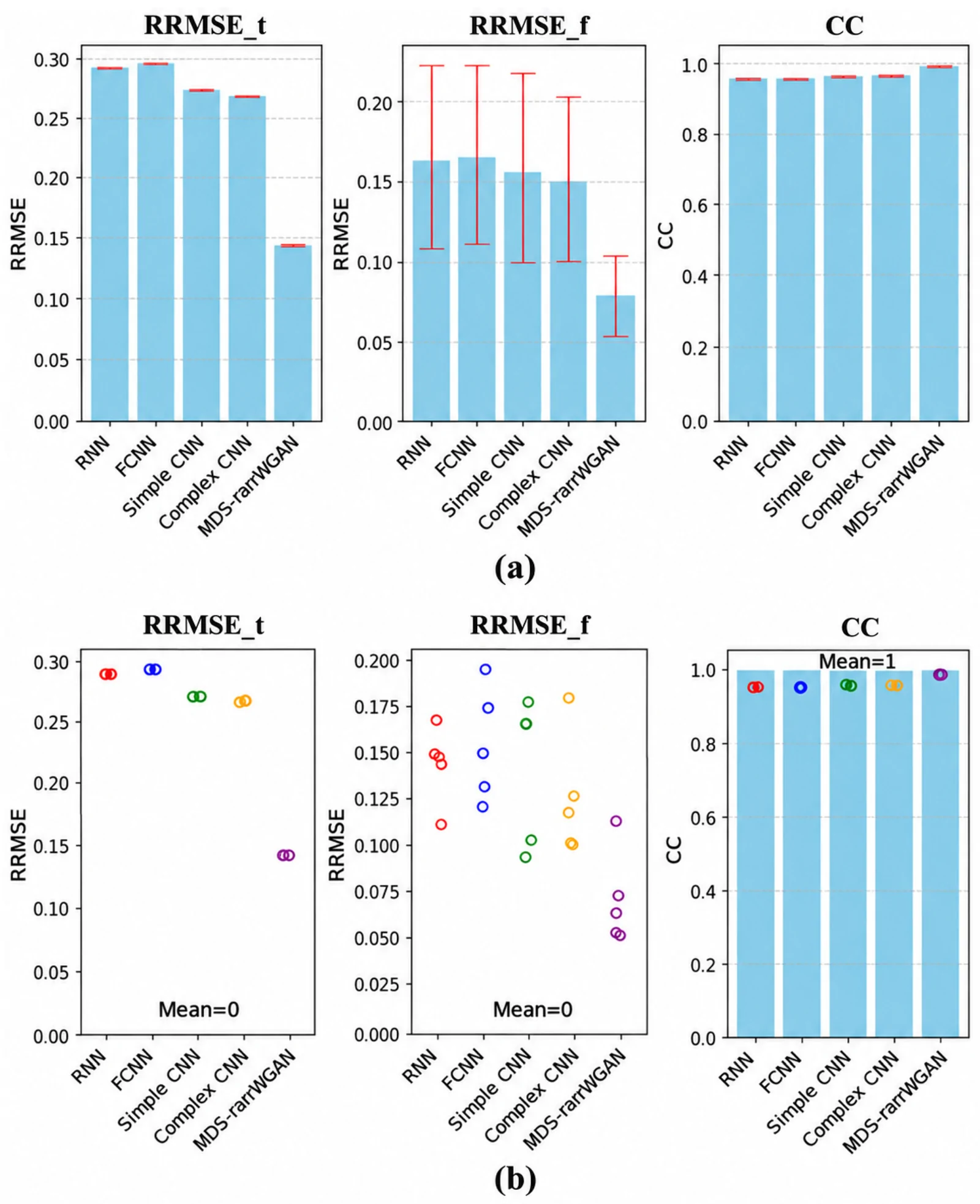
RRMSEs and correlation coefficients of different models for EEG without artifacts: (**a**) without gating; (**b**) with gating.

Figure 7 shows the effect of gating on artifact-free EEG. Without gating, the evaluated models altered these signals to varying degrees. Correctly bypassed segments were copied unchanged to the output, yielding an RRMSE of zero and a CC of one because the input itself served as the reference. The outliers in Figure 7b corresponded to clean segments incorrectly routed to the generator.

## 4. Experiments and Results on the BCI Competition IV 2a Dataset

The preceding experiments used semi-synthetic EEGdenoiseNet mixtures, which provide known clean references and controlled SNRs for evaluating reconstruction performance and the contributions of individual model components. Naturally occurring ocular artifacts vary in waveform, duration, and spatial distribution. To evaluate the proposed method on real motor imagery EEG recordings, experiments were conducted using BCI Competition IV Dataset 2a [29]. The dataset includes three EOG channels and recordings acquired under eyes open, eyes closed, and eye movement conditions. These recordings capture different forms of ocular activity; in this study, EOG artifacts were evaluated collectively. The effect of artifact removal on subsequent motor imagery decoding was also examined.

### 4.1. Evaluation of EOG Artifact Removal on Real Motor Imagery EEG

The models were fine-tuned separately for each participant, following a within-subject adaptation protocol. For each participant, the EEG and EOG blocks from the training session were pooled with the EOG block from the corresponding evaluation session. The resulting 28,644 segments were split in an 8:2 ratio into 22,915 segments for model adaptation and 5729 segments for validation. The 25,344 EEG segments from the evaluation session were not used for adaptation or validation and were reserved for final evaluation. However, because the EOG block from the evaluation session was used during adaptation, this experiment evaluates performance after adaptation to the participant and session rather than generalization to a completely unseen session.

The main training hyperparameters were the same as in the EEGdenoiseNet experiments. After fine-tuning, the gating threshold was selected separately for each participant and kept fixed during testing. For gated-MDS-rarWGAN, the thresholds for the nine participants ranged from −80.2 to 38.9. Because artifact-free EEG ground truth is unavailable for these recordings, ICA-corrected EEG was used as a proxy reference for supervised fine-tuning and quantitative evaluation. ICA was performed using extended Infomax in EEGLAB version 2021.1, followed by automatic component classification using ICLabel version 1.3 and removal of components labeled as “Eye”. Accordingly, the reported RRMSE and CC quantify agreement between the model output and the ICA-derived reference rather than recovery of the unknown true neural signal.

**Figure 8:**
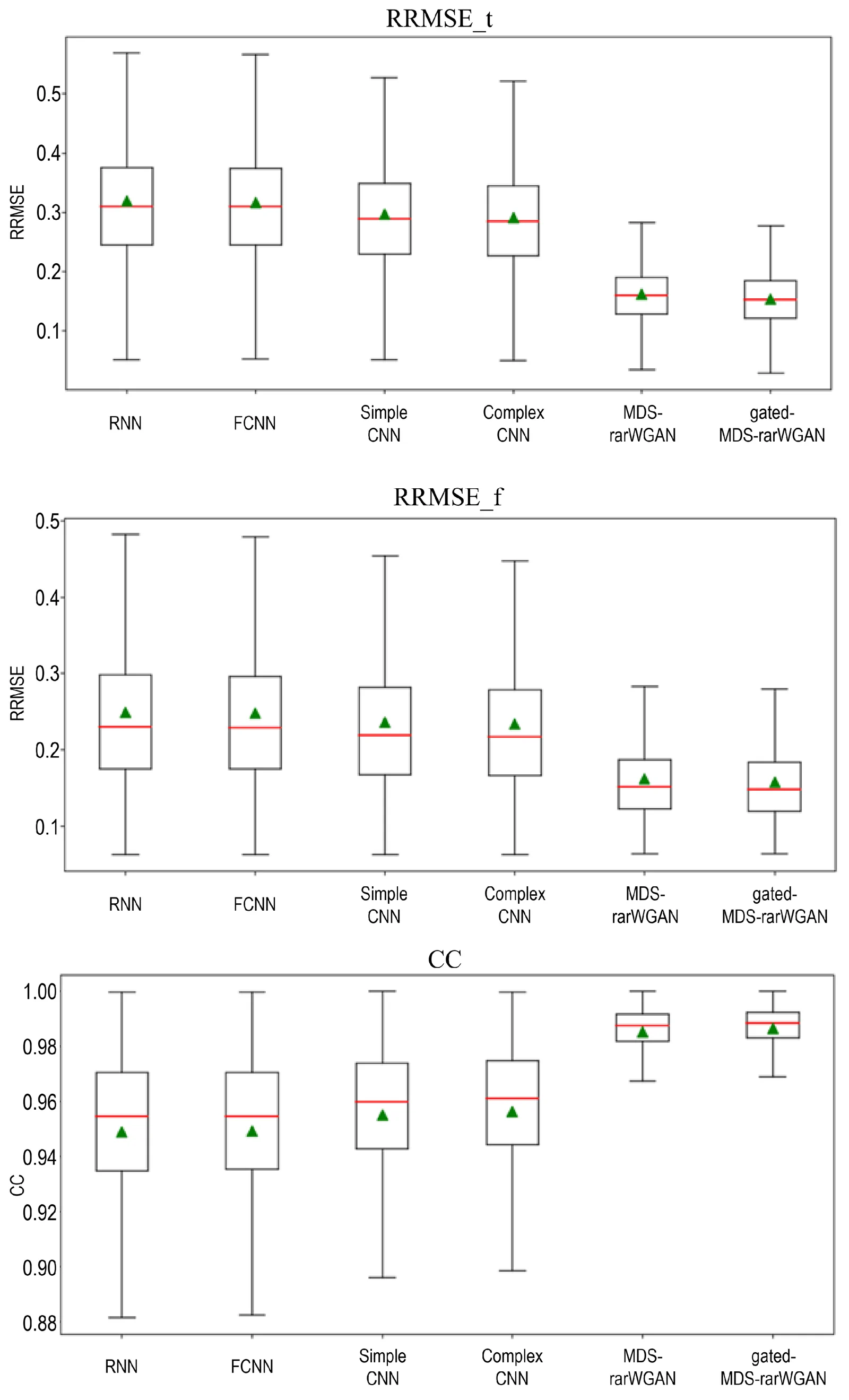
Performance comparison of different methods on the BCI Competition IV 2a dataset.

Figure 8 compares the methods in terms of their agreement with the ICA-derived reference using test data from all nine participants. After fine-tuning, gated-MDS-rarWGAN achieved the lowest time-domain RRMSE (0.154 ± 0.074) and frequency-domain RRMSE (0.158 ± 0.065), and the highest CC (0.986 ± 0.017) among the evaluated deep learning methods.

**Figure 9:**
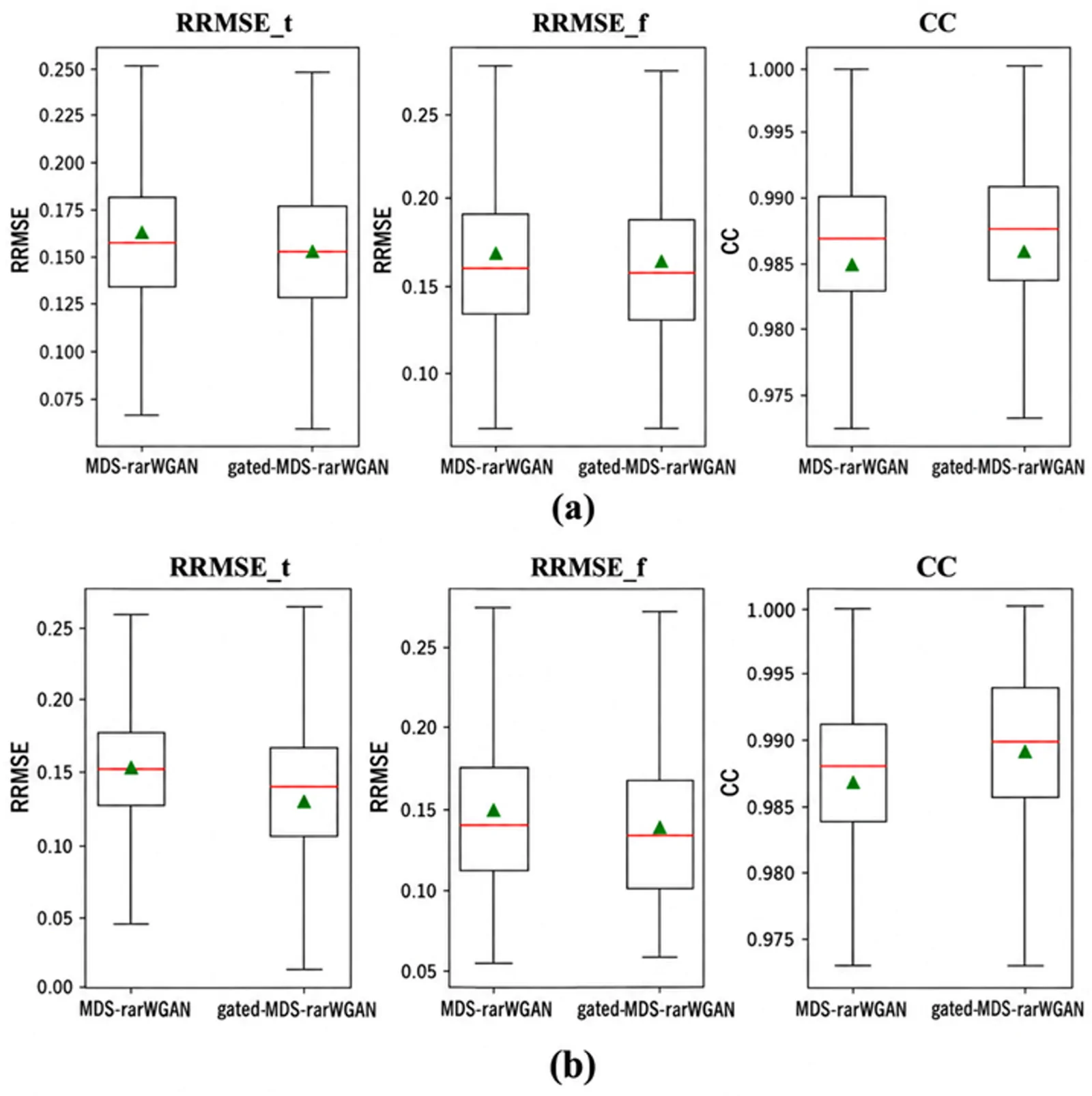
Performance of MDS-rarWGAN and gated-MDS-rarWGAN at different SNR levels: (**a**) low SNR and (**b**) high SNR.

To examine gating effects at different SNR levels, test EEG segments were divided into low-SNR (SNR < 10 dB) and high-SNR (SNR ≥ 10 dB) groups. The 10 dB cutoff was used for this descriptive comparison because most participants had a mean SNR of approximately 10 dB. Here, SNR was calculated relative to the ICA-derived reference and used only to group segments by signal quality for descriptive analysis, rather than to independently determine whether ocular artifacts were present. As shown in Figure 9, MDS-rarWGAN and gated-MDS-rarWGAN achieved similar results across all three metrics in the low SNR group. However, in the high SNR group, gated-MDS-rarWGAN achieved lower RRMSEs in the time and frequency domains and a higher CC than MDS-rarWGAN. These results suggest that gating reduced unnecessary signal changes in the high-SNR group, in which the input signals showed greater agreement with the ICA-derived reference.

**Figure 10:**
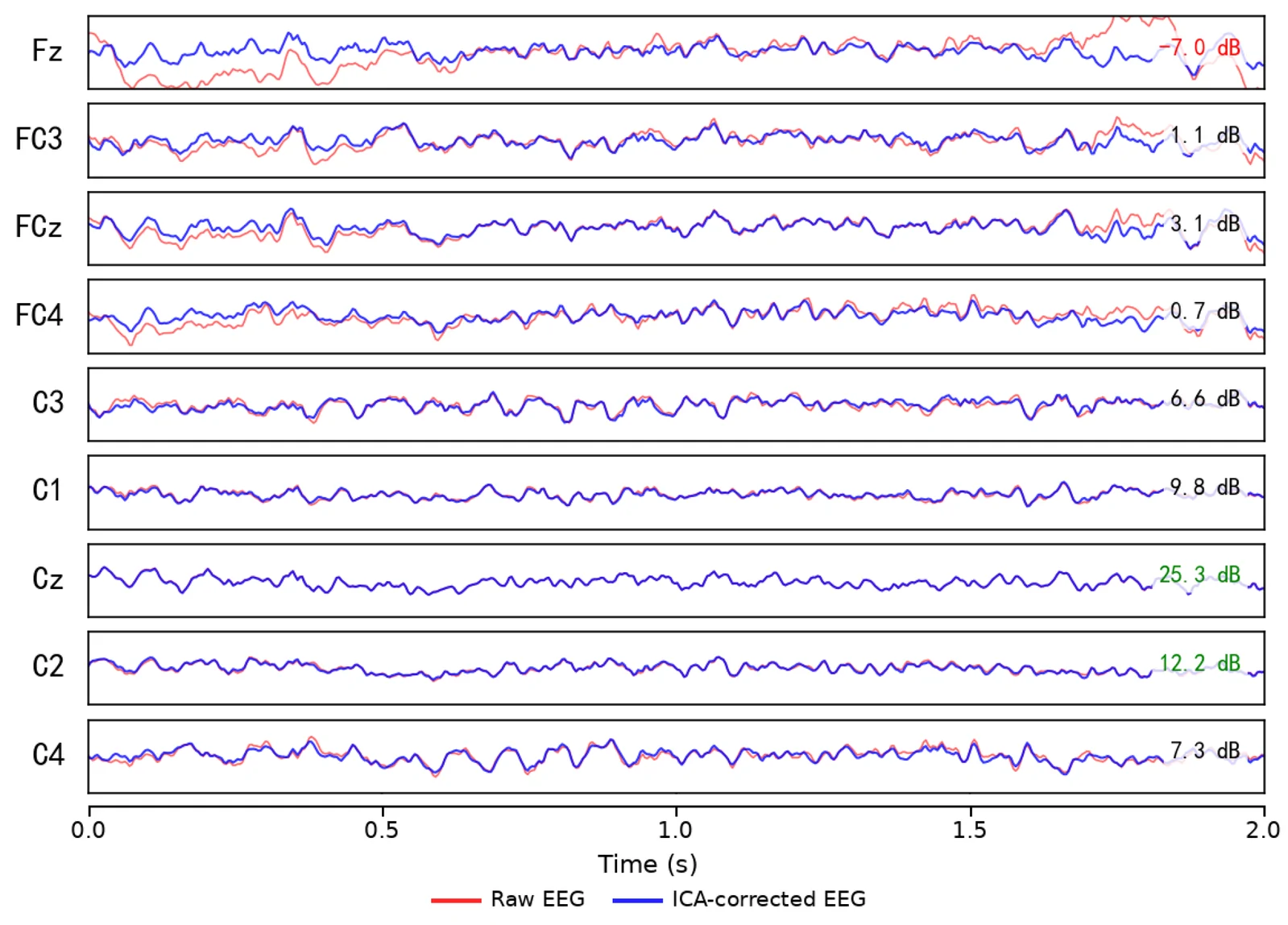
Comparison of raw EEG and ICA-corrected EEG reference signals across nine channels from the BCI Competition IV 2a dataset. SNR labels are colored red for values below 0 dB, black for values from 0 to below 10 dB, and green for values of 10 dB or higher.

**Figure 11:**
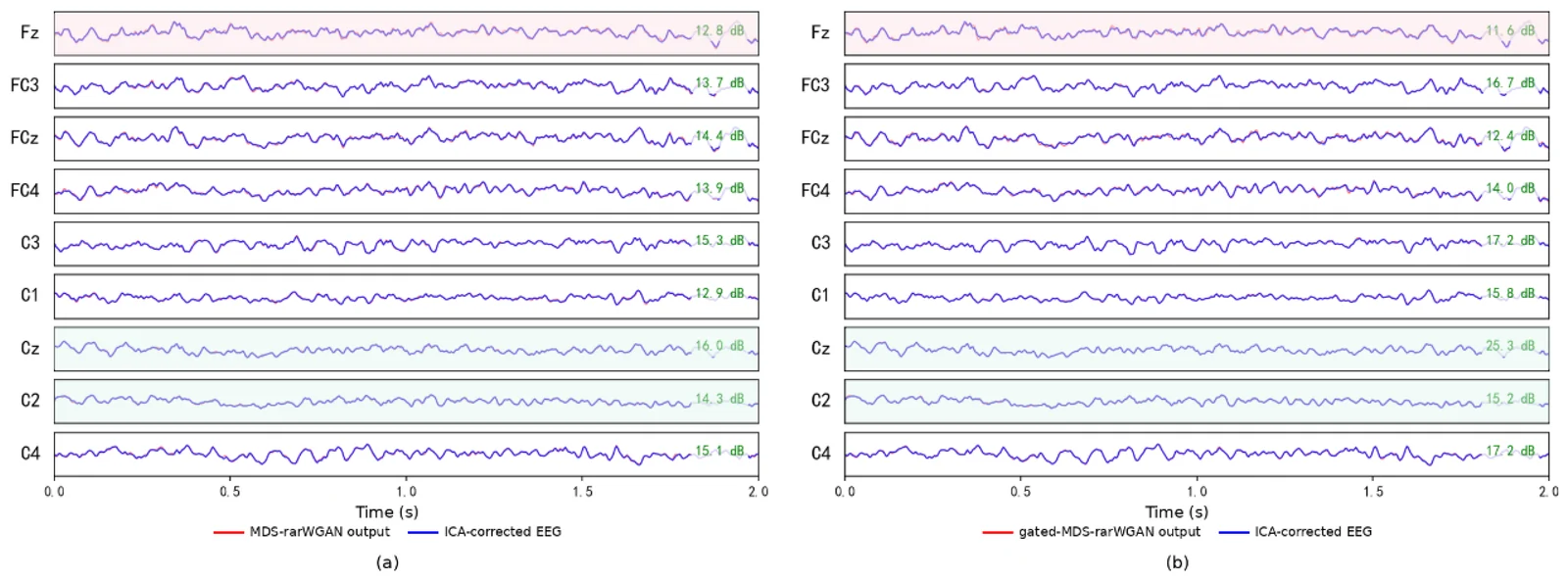
Comparison of EEG signals denoised by the proposed models with the ICA-corrected reference signals. (**a**) MDS-rarWGAN; (**b**) gated-MDS-rarWGAN.

To visualize the waveform differences directly, Figure 10 and Figure 11 present a representative nine-channel EEG segment from Subject 1 in the BCI Competition IV 2a dataset. Figure 10 compares the raw EEG with the ICA-corrected EEG reference. The Fz channel showed the largest deviation from the reference, with an SNR of −7.0 dB, whereas the Cz and C2 channels had substantially higher SNRs of 25.3 and 12.2 dB, respectively.

Figure 11 compares the outputs of MDS-rarWGAN and gated-MDS-rarWGAN with the ICA-corrected reference for the same EEG segment. For the Fz channel, both models increased the SNR relative to this reference, with MDS-rarWGAN yielding a slightly higher value. For channels with original SNR values between 0 and 10 dB, neither model consistently outperformed the other in terms of agreement with the reference. A clearer difference was observed in the Cz channel: MDS-rarWGAN reduced the SNR from 25.3 to 16.0 dB, whereas gated-MDS-rarWGAN retained the original SNR of 25.3 dB and preserved the input waveform.

### 4.2. Effects of EOG Artifact Removal on Motor Imagery Decoding

To evaluate the effects of artifact removal on downstream performance, four-class motor imagery decoding was performed using EEGNet [30]. For each participant, training and validation data were obtained from the training session, whereas evaluation data were obtained from the separate test session. ICA-processed training EEG was divided into training and validation subsets at an approximately 9:1 ratio. Each test set contained 288 trials with 22 channels and 1280 samples per trial. EEGNet was trained using Adam with a learning rate of 0.0002, weight decay of 0.02, a dropout rate of 0.5, and a batch size of 32. Training was limited to 1000 epochs, with early stopping if the validation loss did not decrease for 200 consecutive epochs. Five runs were performed for each participant using different random seeds (42–46). Within each run, the same trained EEGNet model was used to evaluate all eight versions of the test data: raw EEG, ICA-processed EEG, and EEG processed by RNN, FCNN, Simple CNN, Complex CNN, MDS-rarWGAN, and gated-MDS-rarWGAN.

Subject-level paired comparisons used each participant’s five-run mean accuracy, with the nine participants serving as the paired units of analysis. Gated-MDS-rarWGAN was compared with the other seven pipelines using two-sided exact Wilcoxon signed-rank tests, with Holm correction across the seven comparisons. Matched-pairs rank-biserial correlations were reported as effect sizes, with positive values favoring gated-MDS-rarWGAN.

**Figure 12:**
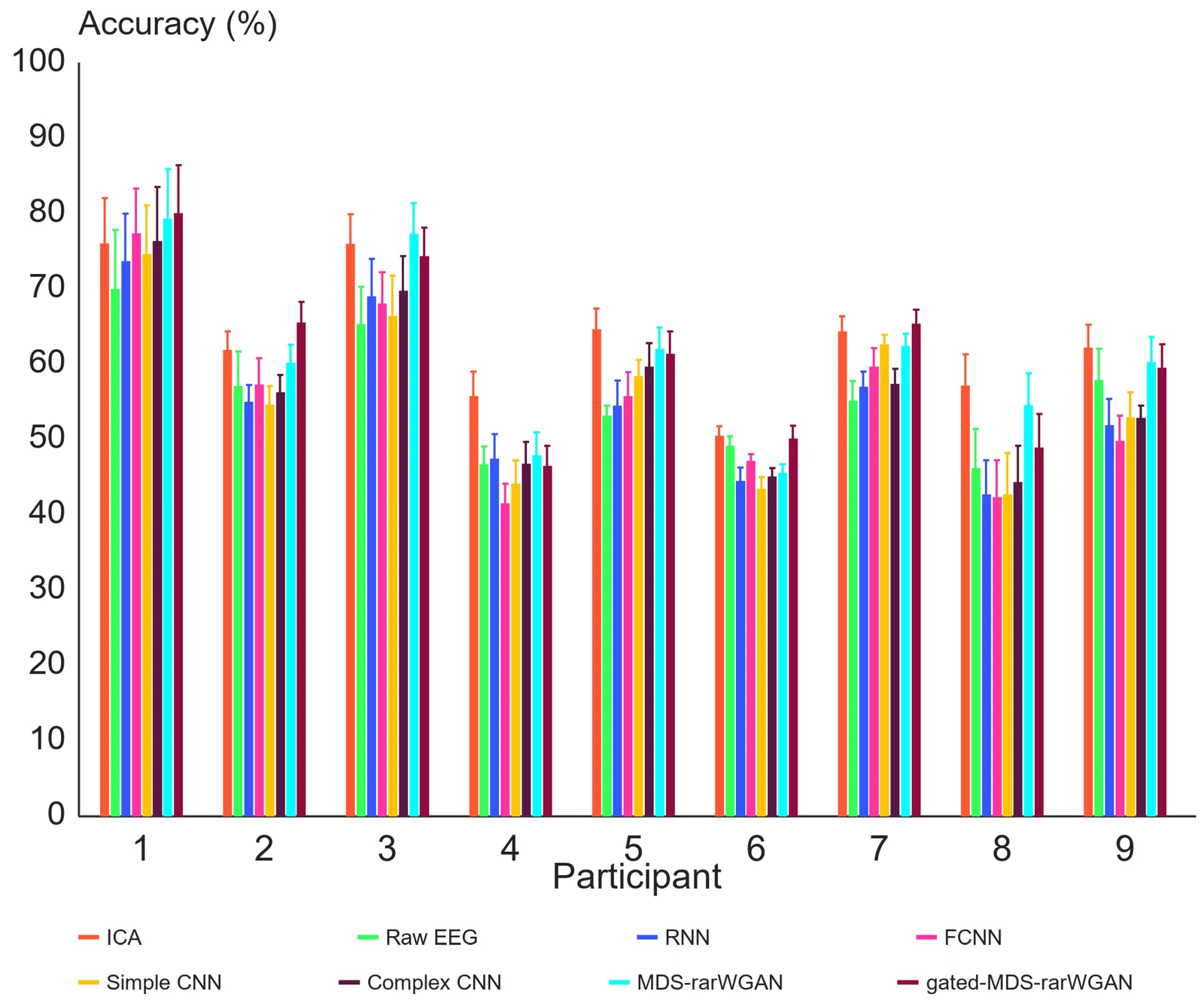
Motor imagery classification accuracies obtained using EEGNet for raw EEG and EEG processed using different artifact removal methods. Bars show the mean accuracy, and error bars indicate the SD across five EEGNet runs for each participant.

Figure 12 presents classification accuracy as mean ± SD across five EEGNet runs for each participant, and Table 5 summarizes the paired comparisons. After Holm correction, gated-MDS-rarWGAN significantly outperformed raw EEG, RNN, FCNN, Simple CNN, and Complex CNN, with mean paired improvements ranging from 4.790 to 6.220 percentage points. Its mean accuracy was 0.249 percentage points higher than that of MDS-rarWGAN and 1.863 percentage points lower than that of ICA, with no statistically significant difference in either comparison.

**Figure 13:**
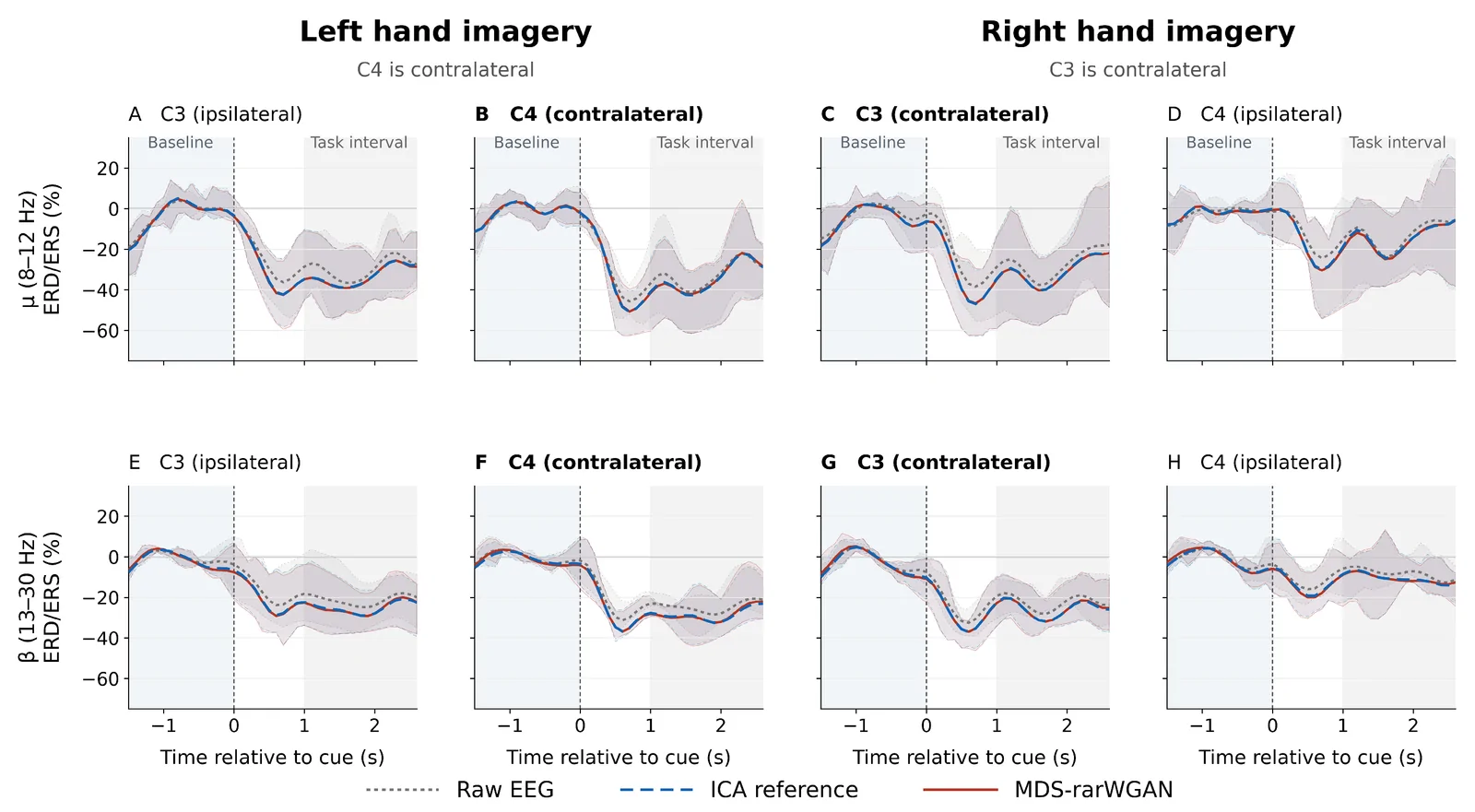
Mu and beta ERD time courses at C3 and C4 during left and right hand motor imagery for raw EEG, the ICA reference, and MDS-rarWGAN. Lines represent means across nine participants, and shaded bands in the corresponding colors indicate the interquartile range (25th–75th percentiles).

To complement the classification results, we compared mu (8–12 Hz) and beta (13–30 Hz) power changes at C3 and C4 during left and right hand motor imagery using raw EEG, the ICA reference, and MDS-rarWGAN outputs. This analysis addresses the preservation of motor imagery-related EEG information alongside artifact attenuation [31,32,33]. All three EEG conditions were evaluated using the same accepted trials. Event-related desynchronization (ERD) was expressed as the percentage change in band power relative to the baseline period (−1.5 to 0 s relative to the cue). Task-period ERD amplitudes were calculated over +1 to +3 s relative to the cue. Figure 13 shows that the mu and beta power changes after MDS-rarWGAN processing closely followed the ICA reference. We further quantified the absolute difference in task period ERD amplitude from the ICA reference at C4 for left hand imagery and C3 for right hand imagery, then averaged the two hand classes within each participant. Compared with raw EEG, the mean amplitude error after MDS-rarWGAN processing decreased from 3.660 ± 3.113 to 0.461 ± 0.321 percentage points in the mu band and from 3.825 ± 2.833 to 0.588 ± 0.326 percentage points in the beta band (mean ± SD across nine participants). These results support the preservation of motor imagery-related mu and beta power changes relative to the ICA reference.

## 5. Discussion

This study proposed gated-MDS-rarWGAN to balance EOG artifact suppression, EEG signal preservation, and computational efficiency. The results indicate that the reconstruction loss and multi-scale and multi-domain feature extraction modules improve signal recovery, whereas the gating mechanism reduces unnecessary processing of EEG segments without evident artifacts. Collectively, these findings highlight the importance of adapting artifact removal to input signal quality rather than uniformly processing all EEG segments.

The adversarial loss encourages the generated signals to match the overall distribution of clean EEG, whereas the mean squared error reconstruction loss directly penalizes pointwise differences between the generated and target signals. This distinction is important because local variations in waveform, amplitude, and phase may contain physiologically relevant information that cannot be adequately preserved through distribution matching alone. The appropriate weight of the adversarial loss may vary with the data distribution, artifact type, and preprocessing procedure and may therefore require adjustment for other datasets.

Previous studies have explored complementary feature representations for EEG reconstruction. DuoCL combines convolutional features at two temporal scales with recurrent modeling [14], while GCTNet combines local CNN features and global Transformer features within an adversarial framework [27]. In the present study, MDS-rarWGAN combines features at multiple temporal scales with frequency-domain information for EEG reconstruction. The ablation results support this design: combining the multi-scale and multi-domain modules reduced the time- and frequency-domain RRMSEs by 23.2% and 32.5%, respectively, compared with rarWGAN. Table 2 further shows that, under the same evaluation protocol, MDS-rarWGAN achieved lower mean RRMSEs in both domains and a higher mean CC than DuoCL and GCTNet. Together, these results support the use of complementary temporal and spectral information for EOG artifact removal on EEGdenoiseNet.

Deep learning denoising models without gating process every input segment and may therefore alter the waveform or spectrum of EEG that contains no evident artifacts. In the proposed method, discriminator scores were used to determine whether an input segment should be processed by the generator or passed directly to the output. The greater benefit observed for high-SNR signals further supports the interpretation that gating helps prevent unnecessary generator-induced changes when the input signals already closely match the reference. Future improvements could incorporate a dedicated artifact-detection branch or soft gating that adjusts the degree of processing according to estimated artifact severity.

Experiments on BCI Competition IV 2a provided a real-data evaluation of the proposed method using ICA-corrected EEG as a proxy reference. After separate fine-tuning for each participant, gated-MDS-rarWGAN showed the closest overall agreement with the ICA-derived reference among the evaluated deep learning methods. MDS-rarWGAN and gated-MDS-rarWGAN showed similar agreement with the reference in the low-SNR group, whereas the gated model showed closer agreement in the high-SNR group.

After Holm correction, the subject-level paired comparisons showed that gated-MDS-rarWGAN significantly improved classification accuracy relative to raw EEG and the four comparator deep-learning artifact-removal methods. The small difference between the gated and nongated models may reflect their shared artifact-removal network. Gating determines whether a segment is processed, so its additional classification benefit may depend on the prevalence and characteristics of artifacts in each participant’s data. Improvements in signal preservation do not necessarily yield a uniform increase in classification accuracy, which also depends on preserving task-discriminative information. The downstream comparison has a limitation: because EEGNet was trained specifically on ICA-processed data, the evaluation may favor test signals whose distribution is more similar to that of ICA-corrected EEG.

The practical significance of the proposed method lies in reducing ocular interference while limiting unnecessary modification of EEG used for subsequent analysis and decoding. Potential applications include EEG research, BCI control, and clinical EEG analysis. The EEGNet results support the method’s usefulness for motor imagery decoding under the present evaluation protocol. Motor imagery-based BCIs for rehabilitation represent one such potential clinical application [2]. Further evaluation in the intended application settings is needed to determine whether the observed benefits translate into more reliable EEG analysis, improved BCI performance, or better clinical outcomes.

This study has several limitations. First, ICA-corrected EEG was used for both supervision and evaluation in BCI Competition IV 2a; the reconstruction metrics therefore measure agreement with this proxy rather than recovery of the unknown artifact-free EEG signal. Adaptation of the denoising model used EOG data from the evaluation session, so generalization to a completely unseen session was not assessed. Subject-independent performance, transfer without adapting the model to target data, and robustness across acquisition systems and natural recording conditions require further evaluation. Second, the method was evaluated for EOG artifacts; future studies should examine other artifact types and mixed contamination. Third, gating operates at the segment level. Independent validation on real recordings requires verified blink and saccade annotations aligned with EOG signals. Further studies should examine discriminator score distributions and the robustness of gating thresholds across participants and contamination levels. The effects of artifact duration and position within a segment should also be examined to guide more localized processing. Fourth, all experiments were conducted offline. Online implementation requires evaluation of system-level latency, computational efficiency, and compatibility with EEG acquisition hardware.

## 6. Conclusions

This study presents gated-MDS-rarWGAN for offline removal of EOG artifacts from single-channel EEG. Reconstruction loss improved waveform recovery, while the multi-scale module captured temporal features at different scales and the multi-domain module integrated complementary information from the time and frequency domains. Based on discriminator scores, the gating unit routed segments classified as artifact-contaminated to the generator and allowed those classified as artifact-free to bypass generator processing. On EEGdenoiseNet, MDS-rarWGAN reduced the time- and frequency-domain RRMSEs by 23.2% and 32.5%, respectively, compared with rarWGAN. Gating also reduced distortion in artifact-free EEG. On BCI Competition IV 2a, after adaptation to each participant and the corresponding evaluation session, the proposed method showed the closest overall agreement with the selected ICA-derived reference among the evaluated deep learning methods. This advantage was more evident in the high-SNR group. Using the same EEGNet classifier, gated-MDS-rarWGAN achieved the highest mean motor imagery classification accuracy among the evaluated deep learning methods, with a mean improvement of 5.672 percentage points over raw EEG. These findings indicate that gated-MDS-rarWGAN balances artifact suppression and signal preservation while retaining information relevant to downstream decoding. Future work will investigate localized soft gating, additional artifact types, cross-subject generalization, and online BCI applications.

## Figures and Tables

**Table 1:** Main hyperparameters of the evaluated models.

Model	Training Epochs	Optimizer	Adam Hyperparameters (*β*_1_, *β*_2_)	RMSprop Decay Rate *β*
FCNN	60	Adam	0.5, 0.9	-
RNN	100	Adam	0.5, 0.9	-
Simple CNN	50	Adam	0.5, 0.9	-
Complex CNN	50	Adam	0.5, 0.9	-
WGAN	50	RMSprop	-	0.9
rarWGAN	50	RMSprop	-	0.9

**Table 2:** EOG artifact removal performance and approximate descriptive effect sizes of different models on the EEGdenoiseNet test set.

Model	RRMSE_t	RRMSE_f	CC	*d*_av_ (RRMSE_t)	*d*_av_ (RRMSE_f)	*d*_av_ (CC)
RNN	0.420 ± 0.093	0.405 ± 0.100	0.898 ± 0.040	−3.82	−3.50	2.15
FCNN	0.375 ± 0.064	0.341 ± 0.065	0.903 ± 0.024	−4.04	−3.43	2.81
Simple CNN	0.350 ± 0.062	0.352 ± 0.055	0.918 ± 0.024	−3.58	−4.12	2.00
Complex CNN	0.339 ± 0.053	0.332 ± 0.047	0.920 ± 0.018	−3.70	−4.03	2.26
WGAN	0.333 ± 0.042	0.317 ± 0.044	0.939 ± 0.017	−4.11	−3.77	1.07
DuoCL	0.342 ± 0.047	0.257 ± 0.031	0.924 ± 0.018	−4.08	−2.55	2.00
GCTNet	0.294 ± 0.033	0.246 ± 0.037	0.944 ± 0.016	−3.44	−1.97	0.76
MSCGRU	0.267 ± 0.027	0.289 ± 0.034	0.941 ± 0.021	−2.84	−3.48	0.82
rarWGAN	0.246 ± 0.025	0.271 ± 0.030	0.944 ± 0.013	−2.15	−3.09	0.85
Multi-scale-rarWGAN	0.230 ± 0.019	0.261 ± 0.023	0.948 ± 0.012	−1.74	−3.12	0.56
Multi-domain-rarWGAN	0.200 ± 0.038	0.188 ± 0.029	0.953 ± 0.015	−0.33	−0.18	0.14
MDS-rarWGAN	**0.189 ± 0.028**	**0.183 ± 0.027**	**0.955 ± 0.013**	**-**	**-**	**-**

Bold values indicate the best performance for each metric.

**Table 3:** Parameter counts and processing times of different models.

Model	Parameters (k)	Mean Processing Time (ms/Segment)
WGAN/rarWGAN	45.19	1.1538
Multi-domain-rarWGAN	46.35	1.3098
Multi-scale-rarWGAN	63.69	1.1930
MDS-rarWGAN	66.90	1.4026

**Table 4:** Comparison of time-domain RRMSE on artifact-contaminated EEG with and without the gating unit.

Model	Without Gating Unit	With Gating Unit	*p*-Value	Holm-Adjusted *p*-Value
RNN	0.420 ± 0.093	0.420 ± 0.090	0.956	1.000
FCNN	0.375 ± 0.064	0.374 ± 0.062	0.901	1.000
Simple CNN	0.350 ± 0.062	0.349 ± 0.059	0.570	1.000
Complex CNN	0.339 ± 0.053	0.339 ± 0.051	0.715	1.000
MDS-rarWGAN	0.189 ± 0.028	0.190 ± 0.026	0.894	1.000

**Table 5:** Subject-level paired comparisons of gated-MDS-rarWGAN with the seven comparator pipelines. Mean differences are expressed in percentage points (pp). Higher/lower indicates the numbers of participants with higher or lower accuracy for gated-MDS-rarWGAN than for the comparator.

Comparator	Mean Difference (pp)	Raw *p*	Adjusted *p* Holm	Rank-Biserial *r*	Higher/Lower
ICA	−1.863	0.4258	0.8516	−0.333	3/6
Raw EEG	+5.672	0.0078	0.0391	+0.956	8/1
RNN	+6.220	0.0078	0.0391	+0.956	8/1
FCNN	+5.856	0.0039	0.0273	+1.000	9/0
Simple CNN	+5.739	0.0039	0.0273	+1.000	9/0
Complex CNN	+4.790	0.0078	0.0391	+0.956	8/1
MDS-rarWGAN	+0.249	1.0000	1.0000	−0.022	4/5

## Data Availability

Publicly available datasets were used in this study. The EEGdenoiseNet dataset is available at https://github.com/ncclabsustech/EEGdenoiseNet (accessed on 10 March 2024), and the BCI Competition IV 2a dataset is available at https://www.bbci.de/competition/iv/download/ (accessed on 7 October 2024). The code for the proposed method is publicly available at https://github.com/liuliu-1110/gated-MDS-rarWGAN (accessed on 14 September 2026).
